# Newly Diagnosed Colonic Adenocarcinoma: The Presenting Sign in a Young Woman with Undiagnosed Crohn's Disease in the Absence of Primary Sclerosing Cholangitis and a Normal Microsatellite Instability Profile

**DOI:** 10.1155/2017/2758769

**Published:** 2017-01-31

**Authors:** Brett Matthew Lowenthal, Ann M. Ponsford Tipps

**Affiliations:** Department of Pathology, University of California, San Diego, 200 West Arbor Drive, San Diego, CA 92103, USA

## Abstract

Ulcerative colitis has long been linked with an increased risk for colonic adenocarcinoma, whereas Crohn's disease (CD) has recently been reported to pose a similar increased risk. We report a 33-year-old healthy female with no family history who presented with abdominal pain and a colon mass. Histopathology revealed a moderately differentiated adenocarcinoma extending through the muscularis propria with metastatic lymph nodes and intact mismatch repair proteins by immunohistochemical expression and gene sequencing. The nonneoplastic grossly uninvolved background mucosa showed marked crypt distortion, crypt abscesses, CD-like lymphoid hyperplasia, transmural inflammation, and reactive epithelial atypia. Additional patient questioning revealed frequent loose stools since she was a teenager leading to diagnosis of a previously undiagnosed CD without primary sclerosing cholangitis (PSC). The adenocarcinoma is suspected to be related to the underlying CD. Newly diagnosed adenocarcinoma in a young female as the presenting sign for CD in the absence of PSC is extremely rare.

## 1. Introduction

Patients with ulcerative colitis (UC), particularly long-standing and extensive disease, have long been established to have an increased risk for developing colonic dysplasia and colonic adenocarcinoma [1–6]. Crohn's disease (CD) is a bit more controversial when it comes to risk of progression to dysplasia and carcinoma. Although colonic adenocarcinoma was first reported as a complication of “regional enteritis” in 1948, now known as CD, this has since only been reported or studied in a limited number of papers [7]. Some studies report that CD poses a similar risk to colonic adenocarcinoma as does UC with similar severity and duration of disease and an increased risk when compared to the general population without inflammatory bowel disease [8–12]. Other studies conclude that there is no association between CD and colonic adenocarcinoma [13–15]. Basseri et al. have recently evaluated screening and surveillance of 411 patients with colonic CD over a 17-year interval and found dysplasia or colonic adenocarcinoma in 5.6% of patients [16].

Inflammatory bowel disease that is associated with primary sclerosing cholangitis (PSC) results in different clinicopathologic parameters as compared to inflammatory bowel disease that is not associated with PSC. Primary sclerosing cholangitis (PSC) is a chronic inflammatory disease that is characterized by mucositis and fibrosis, which causes strictures of the intrahepatic and extrahepatic bile ducts [17]. Sano et al. identified an earlier age at symptom onset, predominantly right-sided disease, and milder symptoms in inflammatory bowel disease patients with associated PSC [17]. We report a 33-year-old woman presenting with colonic adenocarcinoma with normal mismatch repair protein expression and without PSC. The patient was found to have an undiagnosed CD.

## 2. Case Presentation

Our patient is a 33-year-old previous healthy female with no documented past medical history and insignificant family history who presented with severe abdominal pain, nausea, and emesis in July 2012 at an outside institution. A computed tomography (CT) scan of the abdomen revealed thickening of her cecum. She was taken to emergent surgery for what was thought to be a perforated appendix. Intraoperatively, the procedure was switched to a right hemicolectomy due to the presence of a right-sided colon mass.

**Figure 1:**
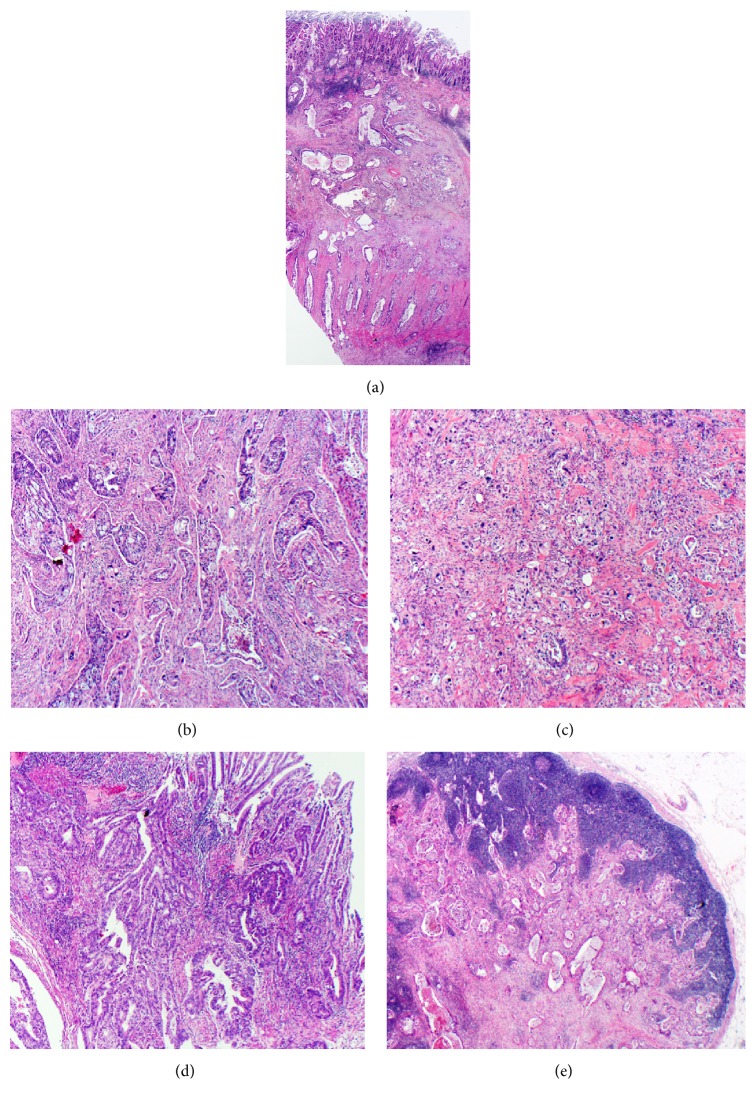
H&E stained sections of grade 2 (moderately differentiated) adenocarcinoma extending through the muscularis propria (pT3). (a) Full-thickness section of the tumor. Adjacent to the tumor and deep to the tumor are lymphoid aggregates composed of tumor infiltrating lymphocytes (20x), (b) areas of tumor highlighting predominantly moderate differentiation (40x), (c) areas of tumor highlighting poor differentiation comprising less than 5% of the overall tumor (40x), (d) tumor originating from surface epithelium (40x), (e) and lymph node metastasis (20x).

Gross pathologic examination of the 14.8 cm in length right hemicolectomy specimen revealed a 7.3 cm cecal mass extending to the ileocecal valve and terminal ileum with no macroscopic perforation. Histopathology revealed a moderately differentiated colonic adenocarcinoma with invasion through the muscularis propria into the subserosal adipose tissue and areas of CD-like lymphoid aggregates adjacent to and deep to the tumor with mild to moderate intratumoral lymphocytes (Figures 1(a) and 1(b)). While Figure 1(c) highlights a focal area of poorly differentiated adenocarcinoma, the tumor has between 50% and 95% glandular architecture designating it as grade 2 moderately differentiated.  Figure 1(d) shows an in situ component of the adenocarcinoma arising from the luminal surface. The tumor did not extend to the serosal surface and the margins of resection were negative. Lymphovascular invasion was present, with metastatic disease found in eight out of the 18 lymph nodes sampled.  Figure 1(e) shows an example of one lymph node with metastatic involvement. No tumor buds or satellite nodules were identified grossly or microscopically.

**Figure 2:**
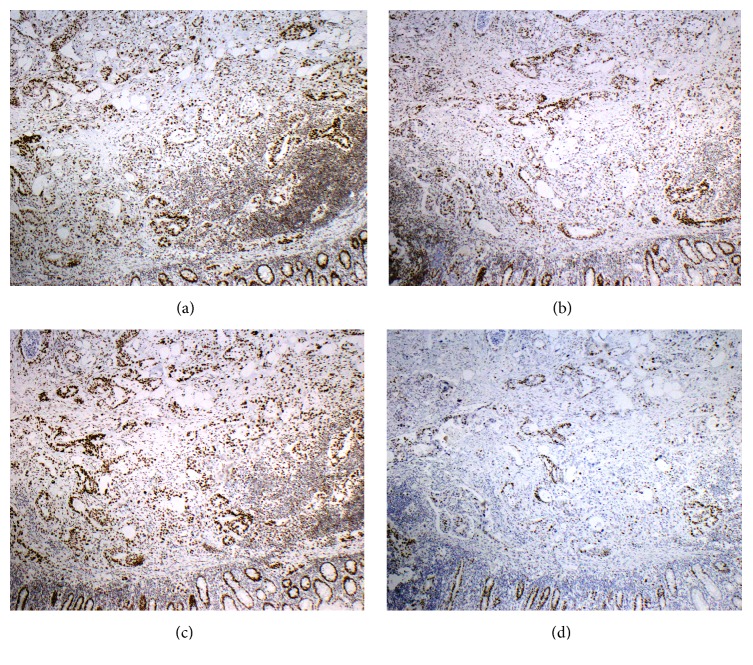
Microsatellite Instability Profile demonstrating no loss of protein expression by immunohistochemical staining. Normal internal control of benign colonic mucosa is located at bottom of each picture. (40x) (a) MLH1, (b) PMS2, (c) MSH2, and (d) MSH6.

Mismatch repair protein (microsatellite instability [MSI]) immunohistochemical stains were performed at the originating outside regional hospital and revealed no loss of MLH1, MSH2, MSH6, and PMS2 protein expression within the invasive tumor cells (Figures 2(a)–2(d)). This is consistent with microsatellite stable colonic adenocarcinoma. Given the patient's young age, right-sided location of carcinoma, and microsatellite stability (by immunohistochemical stains with intact protein expression), HNPCC/Lynch Syndrome (MLH1/MSH2/MSH6/PMS2) Gene Sequencing and Deletion/Duplication testing was performed at ARUP Laboratories (Salt Lake City, UT). This additional full gene sequencing revealed no pathogenic mutations detected in the MLH1, MSH2, MSH6, and PMS2 genes.

The patient then underwent eleven cycles of adjuvant FOLFOX chemotherapy, which was completed in January 2013. A follow-up CT scan of her abdomen in February 2013 showed diffuse colonic thickening, which was concerning for inflammatory bowel disease versus irritable bowel syndrome. Of note, the CT scan revealed no evidence of PSC. In June 2013, a colonoscopy demonstrated patchy erythema, change in vascular pattern, mild friability with no spontaneous bleeding, numerous superficial ulcerations, and granularity in the colon in a patchy distribution. Biopsies revealed cryptitis in the terminal ileum and chronic crypt destructive colitis in the right colon, transverse colon, left colon, and rectum. In July 2013, a positron emission tomography (PET) scan revealed a 1.3 cm paraduodenal lymph node anterior to the inferior vena cava and multiple mesenteric lymph nodes with diffuse uptake. Endoscopic ultrasound-guided biopsy revealed metastatic carcinoma. She was placed on FOLFIRI chemotherapy.

Upon further questioning, the patient reported chronic diarrhea with loose, frequent bloody stools since she was 14 years old. She was referred to the gastroenterology service of our university health system for consultation to confirm her diagnosis of inflammatory bowel disease and plan treatment. The initial right hemicolectomy case was sent to us to evaluate for presence of inflammatory bowel disease, to confirm whether the colitis and chronic diarrhea were a result of inflammatory bowel disease or the chemotherapy regimens.

**Figure 3:**
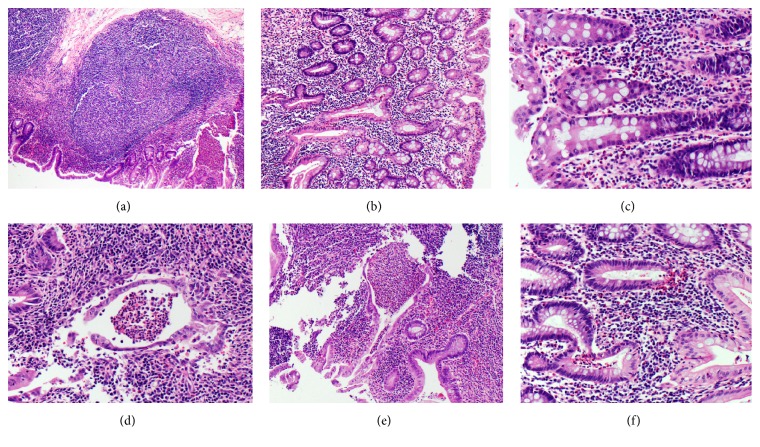
H&E stained sections demonstrating focal chronic-active colitis, consistent with Crohn's disease. (a) Low power view showing dense inflammatory infiltrate composed of lymphoid hyperplasia (center and upper left) combined with transmural neutrophilic inflammation forming deep fissuring ulcer (bottom right) (40x), (b-c) crypt distortion, reactive epithelial atypia with neutrophilic inflammation ((b) 100x, (c) 200x), and (d–f) marked crypt distortion with extensive crypt abscesses in the epithelium ((d) 200x, (e) 100x, and (f) 200x).

Upon our pathologic review of the right hemicolectomy case, the background nonneoplastic colonic mucosa located a few centimeters away from the tumor was noted to show focal chronic-active colitis characterized by marked crypt distortion, crypt abscesses, and reactive epithelial atypia (see Figures 3(a)–3(f), which demonstrate focal transmural neutrophilic inflammation forming a deep fissuring ulcer as well as large hyperplastic lymphoid aggregates). There were no evidence of epithelioid granulomas and no documented upper gastrointestinal tract or perianal disease or fistulizing tracts. The gross description in the outside institution's pathology report did not mention strictures, fat wrapping, serosal adhesions, or cobblestoning of the mucosa. Eight of 18 lymph nodes were positive for adenocarcinoma, but no granulomas or multinucleated giant cells were identified in the nodes. Given the histopathologic findings and the clinical history of unexplained chronic diarrhea for approximately 19 years, the clinicians concluded that the patient most likely had an undiagnosed CD. Calprotectin level and ANCA laboratory tests were not performed, but the patient had elevated C-reactive protein and erythrocyte sedimentation rate levels. As of this report, the patient has no documented evidence of extraintestinal symptoms, such as arthropathy, pyoderma gangrenosum, erythema nodosum, oral aphthous ulcers, episcleritis, or uveitis. She has since completed six cycles of FOLFIRI in March 2014 and is being treated for CD. She has intermittently been seen in clinic for her CD symptoms, which include hematochezia and bowel incontinence. She has had a few colonoscopies demonstrating moderately active ileitis and colitis attributed to her Crohn's disease.

## 3. Discussion

The etiology and pathogenesis of inflammatory bowel disease, including both CD and UC, still remain unknown but are thought to involve a combination of genetic susceptibility, immunity, and disturbances in host responses to intestinal flora [18]. It is the recurrent inflammation with ulceration that increases the risk of cancer in both UC and CD [19]. Despite this current knowledge, it is still controversial whether CD carries the similar risk of colonic adenocarcinoma as does UC. A significant negative in this patient's history was evidence of PSC, as patients with both inflammatory bowel disease and PSC typically have much milder symptoms, right-sided involvement, and younger age at onset. Recent studies have proposed that these patients belong to another phenotype called IBD-PSC independent of UC and CD [20–22]. This proposal has been reinforced with the new discovery of genetic associations with each respective phenotype, including genetic loci of 2p15(REL), 3p21(MST1), and 9q35(CARD9) [23]. Histologic examination of these cases exhibits mild disease, which correlates with the mild symptoms [24].

Colonic adenocarcinoma as the presenting sign in a young woman with undiagnosed CD is extremely rare, especially with no mismatch repair protein expression or associated PSC. This case does follow the typical natural history of CD, where CD is typically diagnosed years before the development of neoplasia although in this case it was diagnosed retrospectively. Colon cancer typically will not present until after many years on surveillance endoscopy and biopsy. After evidence of inflammatory bowel disease was identified within the background colonic mucosa in the adenocarcinoma resection specimen, further questioning by the clinicians revealed a history of chronic unexplained diarrhea with loose and frequent bloody stools for approximately 19 years.

A review of the literature disclosed no case reports of patients presenting with a right-sided colon mass in a patient with undiagnosed Crohn's disease. Imaging and laboratory studies revealed no evidence of PSC. An immunohistochemical stain panel for mismatch repair protein expression (Microsatellite Instability Profile) highlighted presence of all four proteins with no loss of protein expression, confirmed with no pathogenic mutations detected on gene sequencing for the four MSI genes. Thus, HNPCC/Lynch Syndrome is very unlikely, given the combination of a normal mismatch repair protein expression panel, no pathogenic gene mutations, and no family history of malignancy. The etiology of this young woman's colonic adenocarcinoma is suspected to be related to the ongoing inflammation of the previously undiagnosed (and untreated) inflammatory bowel disease.

This case highlights the necessity to scrutinize the “uninvolved” sections of colonic mucosa in colon cancer cases for evidence of inflammatory bowel disease. Not only may it provide supplemental information to account for symptoms, but it can influence treatment and surveillance regimens after the carcinoma has been treated with surgery and chemotherapy.
